# Editorial: Novel approaches and concepts of biomarker discovery for cancer

**DOI:** 10.3389/fgene.2022.955062

**Published:** 2022-08-24

**Authors:** Guini Hong, Wan Li, Xiaopei Shen

**Affiliations:** ^1^ School of Medical Information Engineering, Gannan Medical University, Ganzhou, China; ^2^ College of Bioinformatics Science and Technology, Harbin Medical University, Harbin, China; ^3^ Fujian Key Laboratory of Medical Bioinformatics, Department of Bioinformatics, School of Medical Technology and Engineering, Fujian Medical University, Fuzhou, China

**Keywords:** cancer, biomarker, sequencing, microarray, PCR-based technology, computational analysis

DNA sequencing, microarray technology, and PCR-based techniques provide sensitive and specific methods to explore biomarkers for cancer diagnosis, prognosis, and treatment. The manuscripts in this Research Topic reflect the efforts of the most relevant research groups in the scientific field of cancer biomarkers.

The increasing availability of gene expression data generated by high-throughput genome research makes it possible to identify RNA characteristics potentially associated with cancer. Dong et al. proposed an algorithm named mRBioM to identify potential mRNA biomarkers for gastric adenocarcinoma (GA) using transcriptomic data from The *Cancer* Genome Atlas (TCGA). Unlike existing algorithms, mRBioM evaluates the potential of each differentially expressed mRNA as a biomarker by combining the corresponding amount of information at the transcription and protein levels based on the information entropy theory. It was concluded that the identified biomarkers might have good application prospects for early diagnosis of GA and help to elucidate the mechanism governing the occurrence and development of GA. Additionally, mRBioM was expected to be applied to identify candidate biomarkers for other types of cancer. Yi et al. analyzed Triple-negative breast cancer (TNBC) data from TCGA and Gene Expression Omnibus (GEO) databases. They presented an accurate prognostic evaluation tool based on immunophenotyping of TNBC patients.

Unlike the analysis of whole genomes, another class of cancer research either focuses on an individual gene or evaluates cancer biomarker potential for a certain type of functional genes. For example, Zhong et al. revealed that Syndecan-1 regulated immune infiltration by influencing T Cells in Glioma at the transcriptome level. They also identified eight critical genes related to SDC1 and immune infiltration in glioma and evaluated their clinical significance by survival analysis. In addition, based on a set of 74 genes associated with cholesterol homeostasis, Chen et al. constructed and validated a cholesterol homeostasis-related gene signature to predict endometrial cancer prognosis by analyzing endometrial cancer patients’ gene expression data from TCGA.

PCR-based technology is one of the most widely used techniques in molecular biology. The paper contributed by Zhou et al. applied the real-time quantitative PCR to measure the circulating cell-free mitochondrial DNA (ccf-mtDNA) content in a hospital-based cohort of hepatocellular carcinoma (HCC) patients. They reported that higher content of ccf-mtDNA was significantly associated with worse overall survival of patients. The finding of this manuscript provided a potential non-invasive biomarker to stratify HCC patients who might benefit from transarterial chemoembolization combined with traditional Chinese medicine treatments. Furthermore, based on PCR technology, Sun et al. confirmed that the expression of HKII and HIF-1α was associated with the progression and differentiation of prostate cancer, indicating that HKII and HIF-1α might be novel prostate cancer biomarkers with potential clinical application value.

Epigenetic changes play an essential role in the initiation and development of cancer. The methylation modification is the commonest epigenetic alternation and a type of promising biomarker in cancer patients. The review of Zhang et al. mainly focused on describing the current progress and future direction of DNA or RNA methylation modification detection in cancer with the cutting-edge nanopore sequencing approaches. They also addressed the CRISPR/Cas9-targeted enrichment nanopore sequencing method and its application in discovering epigenetic changes in cancer, highlighting the advances of nanopore sequencing techniques for detecting methylation modifications and biological discoveries with their application in the context of cancer.

The advances in (epi)genomics, transcriptomics, proteomics, metabolomics, and immunomics and the development of novel bioinformatics/computational methods and tools have improved the approach to studying complex diseases such as cancer. The method article on this Research Topic proposed an algorithm for identifying potential mRNA biomarkers from complete transcriptomic profiles of cancer. The research articles mainly focused on cancer survival analysis, and three of them analyzed tumor immune infiltration. One article investigated the serum as a promising non-invasive surrogate for solid tissue to study disease-associated molecular biomarkers. Finally, the review article looked at recent advances in nanopore sequencing applications. It is anticipated that this Research Topic of Frontiers in Genetics will be of great value to many investigators, and it will inspire new and transformative multi-omics research in the realm of cancer biomarkers.

